# Role of short interpregnancy interval, birth mode, birth practices, and the postpartum vaginal microbiome in preterm birth

**DOI:** 10.3389/frph.2022.1082199

**Published:** 2023-01-04

**Authors:** Nkechi Martina Odogwu

**Affiliations:** ^1^ Department of Pediatrics, University of Minnesota Medical School, Minneapolis, MN, United States; ^2^ School of Public Health, University of Minnesota, Minneapolis, MN, United States

**Keywords:** short interpregnancy interval, birth mode, vaginal microbiome, postpartum infections, preterm birth, vaginal cleaning, caesarian delivery, vaginal delivery

## Abstract

There have been widely documented beneficial role of vaginal Lactobacillus species as an important biomarker for vaginal health and healthy pregnancy progression. When translating this to clinical settings, pregnant women with low proportions of Lactobacillus and commensurately high proportion of rich and highly diverse abnormal microbiota are most likely to encounter negative pregnancy outcome such as preterm birth and postpartum complications. However, multiple literatures have also addressed this notion that the absence of a Lactobacillus-dominated microbiota does not appear to directly imply to a diseased condition and may not be a major determinant of negative obstetric outcome. Caesarian delivery is notably a risk factor for preterm birth and postpartum endometritis, yet recent data shows a trend in the overuse of CS across several populations. Growing evidence suggest the potential role of vaginal/uterine cleaning practice during CS procedures in influencing postpartum infections, however there is a controversy that this practice is associated with increased rates of postpartum endometritis. The preponderance of bacterial vaginosis associated bacteria vagitype at postpartum which persist for a long period of time even after lochia regression in some women may suggest why short interpregnancy interval may pose a potential risk for preterm birth, especially multigravidas. While specifically linking a community of microbes in the female reproductive tract or an exact causative infectious agent to preterm birth and postpartum pathologies remains elusive, clinical attention should also be drawn to the potential contribution of other factors such as short interpregnancy interval, birth mode, birth practices and the postpartum vaginal microbiome in preterm birth which is explicitly described in this narrative review.

## Introduction

The objective of the Human Microbiome Project (HMP) is to provide a detailed characterization of the structure and composition of the microbiome across various body habitats (1). About a decade after the inception of HMP, there has been an increasing interest in understanding the composition and function of the vaginal microbiome (VMB) and its contribution to pregnancy outcome. As recommended by HMP, DNA sequencing-based interrogation of the microbiome has provided better insight into the myriads of microbes inhabiting the vaginal econiche (2, 3). This approach has also been deployed in several vaginal microbiome studies (4, 5). From an ecological standpoint, it is well documented that a robust, *Lactobacillus*-dominated vaginal profile are considered optimal for healthy pregnancy outcomes whereas an increased diversity and richness of vaginal microbial community with high proportions of non-*Lactobacillus* dominated microbiota including several bacteria vaginosis associated bacteria (BVAB) are considered unhealthy and a potential risk factor for preterm birth (6), postpartum endometritis (7) and preterm premature rupture of membranes (8, 9). Hitherto, there has been several conflicting reports regarding the role of the vaginal microbiota in determining obstetric outcome. While some studies suggest a role for the vaginal microbiota in adverse pregnancy outcome such as preterm birth (10–14) and postpartum complication (15). Other studies suggests that the presence or lack of *Lactobacillus* vagitype may not be requisite for adverse pregnancy outcomes (16, 17). The maternal vaginal microbiome also plays an essential role in the initial colonization of new-born babies’ microbiota and therefore the development of a healthy microbiota (18). Aside this, growing evidence also suggest that birth mode [Cesarean section (C-section) or vaginal delivery] play a critical role in the initial colonization of the infant microbiome and may be associated with long-term health outcomes (19). Short interpregnancy intervals also appear to be associated with increased risks for adverse pregnancy outcomes for women of all ages (20). All these factors are holistically drivers of the preterm birth cascade and are describe in this review. Here we examine the role of short interpregnancy intervals, mode of delivery, birth practices and the postpartum microbiome in preterm birth to emphasize the need to track and pinpoint other potential drivers of preterm birth and to provide a basis to recommend targeted strategies for restoring the reproductive health fitness of women of reproductive age.

## Methods

### Literature search and review procedures

**Figure 1:**
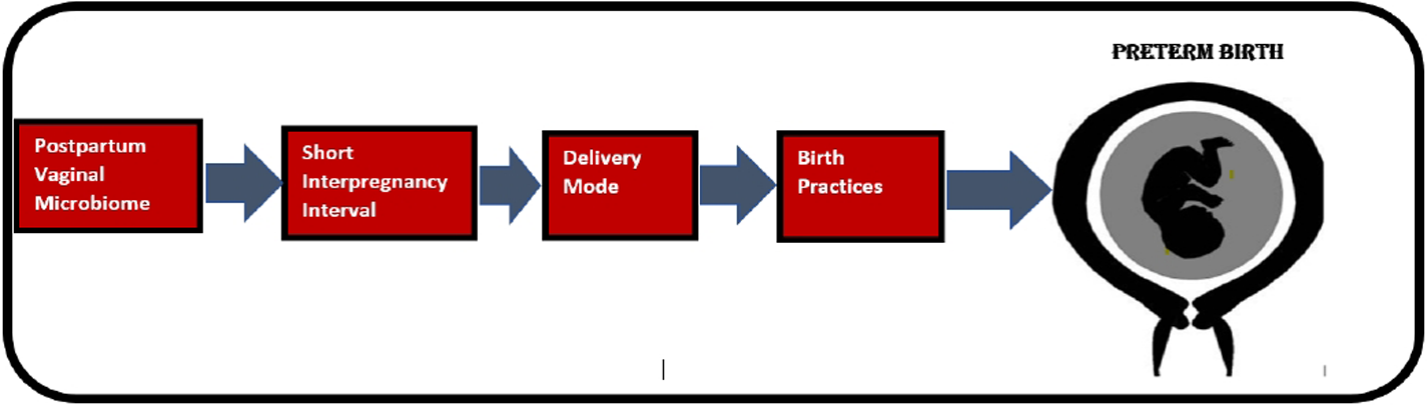
Causal chain diagram depicting some underlying factors that contribute to preterm birth.

Literature search was conducted in PubMed, Web of Science, Embase and Cochrane Library using multiple keywords both alone as well as in combination so that all factors that have received recent scientific attention is included. Birth mode, postpartum, vaginal microbiome, postpartum infections, preterm birth, vaginal cleaning, caesarian delivery, vaginal delivery, uterine cleaning, postpartum endometritis were important key words used. Peer-reviewed, English-language original study articles published in key papers addressing whether a given factor plausibly contributes to PTB were included. When selecting articles addressing vaginal preparation pre-CS, articles on WHO recommendations, randomized control trials and other articles supported by Cochrane review were included. Articles addressing similar factors contributing to preterm birth were excluded by focusing on more recent articles, web-based material were also excluded except from scientifically trustworthy sources [e.g., World Health Organization (WHO)]. All results were uploaded to EndNote for the literature selection. The reference lists of eligible articles were also screened to detect relevant articles that were not identified by the initial search strategy. As illustrated, Figure 1 depicts a causal chain diagram addressing some underlying causes of preterm birth.

### Vaginal microbiome and preterm birth

Preterm birth (PTB), defined by the World Health Organization (WHO) as the delivery of an infant before completed 37 weeks of gestation. PTB represents one of the major challenges facing obstetrics and a leading cause of neonatal mortality worldwide (21). Annually an estimated 15 million babies are born too early and approximately 1 million children die each year due to complications of preterm birth (22). Many of the causes of PTB are incompletely understood since more than 65%–75% spontaneous PTBs occurs with idiopathic onset (23, 24), thus clinical symptoms alone are not enough to predict or identify women at higher risk of PTB. PTB is common in certain maternal or fetal conditions, such as preeclampsia, intrauterine infection, cervical insufficiency, preterm premature rupture of membranes (PPROM), polyhydramnios, and fetal malformations (25). Previous miscarriage, multifetal gestation, young or advanced maternal age, assisted reproductive technology (ART), black race, smoking, extremes of body-mass index (BMI), and low socioeconomic status are known risk factors for PTB (23, 24). Disruption of the vaginal microbiota (vaginal dysbiosis) which are often asymptomatic in most women is notably a risk factor for PTB (26–28). As an illustration, prior research has demonstrated that bacteria activate the innate immune system of the reproductive tract and fetal membrane driving an inflammatory cascade that results in cervical remodeling and disruption of fetal membrane architecture resulting in PTB (29–32). Additionally, *in utero* microbial colonization of fetus has been suggested to play a substantial role in the early establishment of the infant's microbiome (18, 33). Conversely there have been other conflicting reports regarding the association of bacteria diversity with preterm birth outcomes. A longitudinal analysis of the vaginal microbiome in patients that encountered preterm birth revealed no difference in bacterial taxa associated with spontaneous preterm birth and term delivery (16). This report was further reinforced by the study of Elovitz and others (17) which addressed the notion that a non-*Lactobacillus* dominated flora may not be requisite for adverse pregnancy outcomes as some women with high level of vaginal bacterial diversity in this cohort delivered at term (17). *L. crispatus* is considered an optimal vaginal microbiome that supports healthy pregnancy progression (34), yet a Japanese cohort found L. *crispatus* as predominant microbes associated with women who delivered preterm (35). This inconsistencies and conflicting reports across studies are largely ethnically dependent and may have been reached due to variations in patients characteristics across various study cohorts; the definitions of PTB across low, low-middle- and high-income country; different cohort selection criteria (inclusion, exclusion, and elimination criteria) and diverse study designs, including different sequencing technologies and different 16S regions sequenced. Therefore, current research on microbiome and pregnancy should focus on better understanding of the dynamics within different cohorts/patients, as patient characteristics vary from study-to-study. Additionally, the role of the vaginal microbiome in the etiology of PTD remains to be delineated as such more attention should be drawn to the other potential factors that may contribute to preterm birth.

### Role of birth mode in preterm birth

The maternal microbiome plays an essential role in the initial colonization of new-born babies and therefore the development of a healthy microbiota (18). Thus, the birthing process is a significant event in the initial seeding of the infant microbiome. Recent studies suggest that birth mode [Cesarean section (C-section) or vaginal delivery] is crucial in the initial colonization of the human microbiome and may be associated with long-term health outcomes (19). Immediately after birth, newborn babies experience rapid microbial colonization from their mothers and the surrounding environment (18), as such maternal Bacteroides strains, and opportunistic pathogens associated with the hospital environment are most likely transmitted to babies delivered by caesarean section (36, 37). To further corroborate this, a recent cross-sectional pilot study examined the association between a woman's birth mode and her vaginal microbiota in adulthood and found that women with low relative abundance of *Lactobacillus* spp. or women lacking a vaginal Lactobacillus profile had 3-fold increased odds of being born *via* C-section, indicating that C-section is associated with vaginal dysbiosis in adulthood (19). Further, an observational study across 10 different cohorts comprising of 1,033,3501 women demonstrated that pregnant women with history of previous cesarean section are significantly at a higher risk of preterm birth in subsequent births (38). To circumvent the incidence of preterm birth especially in women with history of Caesarian delivery the frequency of unnecessary cesarean section when there is no medical need should reduce. Health care system should also optimize the use of CS since underuse of CS may lead to maternal and perinatal mortality and morbidity and overuse of CS may create harm and potentially predispose to preterm birth.

### Vaginal microbiome and postpartum infections

Infections at postpartum presents a significant burden to the hospital system and remains a significant cause of maternal mortality and morbidity worldwide (39, 40), yet the epidemiology of several post-delivery pathologies including postpartum endometritis, postpartum sepsis and chorioamnionitis remains elusive and has not been well characterized. Postpartum endometritis (PPE) describes an inflammation or infection of the upper genital tract comprising the endometrium, myometrium, and the surrounding tissue. It typically involves multiple infectious agents ranging from a mixture of strict/facultative anaerobes to aerobes from the reproductive tract. Early postpartum endometritis occurs within (1–2 days) and infection that persists for more than 2 days are considered late postpartum endometritis (7, 41, 42). Several syndromes including fever, abdominal swelling, foul smelling lochia discharge, uterine tenderness, pain in the pelvis & lower abdomen, constipation, abnormal vaginal discharge, and vaginal bleeding are associated with postpartum endometritis (43–45). A global estimate of maternal death caused by several post-delivery infections reports a higher prevalence in countries with low-resources compared to high-resource settings (46, 47). Caesarian delivery is one of the major risk factors for PPE (40, 41). Prior research has shown that CS delivery introduces BVAB through surgical sites which follows a descending pathway into the vagina thus accentuating PPE (48) Consistent with this, Rosene and others (49) demonstrated that results from swabs and endometrial culture isolated from the genital tract of women with early postpartum endometritis predominantly comprises of microbes associated with vaginal dysbiosis including Mycoplasma, *Chlamydia trachomatis*, *Gardnerella vaginalis*, Peptococcus spp., Bacteroides spp., *Staphylococcus epidermidis*, group B Streptococcus, *Ureaplasma urealyticum* (49) *Clostridium sordellii* (50), *Clostridium perfringens*, streptococcal toxic shock syndrome (51) and *Leptrotrichia amnionii* a recently emerging pathogen of postpartum endometritis (52). DNA sequencing-based interrogation of the vaginal microbiome has vastly been studied in post-natal women (53–60). These studies have shown that a dramatic switch to a vaginal bacterial community lacking in *Lactobacillus* species is common after pregnancy (53–60). Although, majority of the aforementioned studies on the postpartum vaginal microbiome explored vaginal samples collected at 6 weeks postpartum (53, 54) and 1-week post-delivery (59), thus early postpartum microbiological content which could be potential biomarkers of early PPE are not accounted for. One potential way by which postpartum infections can be alienated is thorough screening of women for bacterial infection at late trimester. As an illustration, a recent study across a cohort of 61 Caucasian pregnant women at the first and third trimester of pregnancy investigated the distribution of *Gardnerella vaginalis* clades in the vaginal ecosystem and found a correlation between *Gardnerella vaginalis* clades and the whole vaginal microbiome (61). *Gardnerella vaginalis* is notable for been responsible for a significant number of infections in postpartum women worldwide and a leading cause of preterm birth and neonatal infection. Therefore, treating women with bacterial vaginosis at late trimester and screening asymptomatic women for bacterial vaginosis at late trimester should be considered to reduce the risk of PPE. To improve our understanding of the underlying epidemiology of PPE, future study should therefore consider early collection and investigation of swab samples few hours before and after surgical procedure (CS) or immediately after delivery especially for women with history of PPE.

### Role of birth mode and birth practices in postpartum infections

There are multiple risk factors for postpartum endometritis including delivery mode (62), early and moderately preterm birth (63, 64), BMI (65), bacterial colonization with group A or group B *Streptococcus* (66), chorioamnionitis, prolonged rupture of membranes, prolonged operative time, poor nutrition, bacterial vaginosis, and multiple vaginal examination (67, 68). Of these risk factors associated with PPE, there are now substantial evidence linking the association of birth mode (CS or vaginal delivery) with PPE (40, 41). A Cochrane database review focused on understanding the etiology of endometritis after delivery demonstrated that postnatal women face a nearly 5–10 times greater risk of PPE following caesarean section compared to vaginal delivery (69). The prevalence of PPE is also higher in planned or emergency caesarean sections (1.5%–5%) compared to a lower prevalence (1.2%–2%) in vaginal deliveries (7, 70). Additionally, postpartum endometritis is primarily prevalent in 27% of cesarean deliveries and only 1%–3% of vaginal births (44, 62). PPE risks are also higher following caesarian deliveries performed in labor (3%–11%) compared to prelabor caesarian deliveries (0.5%–5%), as well as in patients with subsequent ruptured membranes compared to intact membranes (3%–15% vs. 1%–5%, respectively) (71). Caesarean section (CS) continues to evoke worldwide concern because of their steady increasing rates reaching up to 40.5% birth globally (72). According to new research from the World Health Organization (74, 75), caesarean sections now account for more than 1 in 5 (21%) of all childbirths. This number is set to continue increasing, with nearly a third (29%) of all births likely to take place by caesarean section by 2030 (73). While a caesarean section can be an essential and lifesaving surgery, it can put women and babies at unnecessary risk of short- and long-term health problems if performed when there is no medical need. With CS as a critical risk factor for PPE and with several literature reports of PPE potentiated by bacterial infections (76, 77), an important investigation of what practices are performed during CS surgical procedure could lends clue to the pathogenesis of PPE. PPE mediated by CS appears to be ethnically dependent. A recent analysis confirms the increasing trend of CS in all regions at different pace. Subregions with the greatest increases were Latin America/Caribbean, Eastern Asia, Western Asia, Northern Africa (42.8-, 44.9-, 34.7- and 31.5%-point increase, respectively) while sub-Saharan Africa and Northern America (3.6- and 9.5%-point increase, respectively) had the lowest rise (74, 78, 79). This variation in the frequency of occurrence of postpartum endometritis mediated by CS across ethnically diverse groups in various populations may partly be due to variation in clinical settings, differences in individual or patients’ characteristics especially in the context of susceptibility to infections and practices performed during CS procedures (79). While Lyon and Richardson (80) are of the school of thought that postpartum morbidities could be reduced by modifications in surgical technique (80). Epstein (81) is of the opinion that an alteration or modification in modern-day birthing surgical procedure has the potential of introducing new microbes that are non-susceptible to current antimicrobial therapy (81). In keeping up with this, there have been a lingering hypothesis that the practice of vaginal cleansing/vaginal preparation with vaginal antiseptic such as povidone-iodine and chlorohexidine before Cesarean delivery could reduce the rate of post-cesarean endometritis (82). Although the aforementioned trend analyses showed the underuse of CS in low-middle-income countries (especially sub-Saharan Africa), yet women in low, low-middle income countries appear to be more susceptible to postpartum infections caused by *E. coli*, *Proteus* spp., *N. gonorrhoea*, Group A and Group B *Streptococcus* and other strict Facultative anaerobes compared to women in high-income countries (HIC) (66, 83). The high incidence of postpartum endometritis in low-income settings compared to high income settings (83) may partly be due to the practice of not cleaning the vagina with cleaning antiseptics before cesarean delivery compared to high income settings where this practice is frequently performed. Support for the potential role of vaginal/uterine cleaning practice during CS procedures in influencing PPE comes from several reports showing that vaginal preparation or cleansing with povidone-iodine or chlorhexidine solution before cesarean delivery significantly reduced the incidence of post-cesarean endometritis (82, 84–86). A 2018 Cochrane Database systematic review published a study from a North American population demonstrated that that vaginal preparation or vaginal/uterine cleaning with povidone-iodine or chlorhexidine solution before cesarean delivery significantly reduced the incidence of post-cesarean endometritis (84). This observation is further corroborated in the study of (86, 87) which reported similar findings. Conversely, a randomized clinical trial across 648 patients (uterine cleaning group 336 patients and no cleaning group (312 patients) demonstrated that uterine cleaning after delivery of the placenta during CS can be omitted as a surgical step during the operation as cleaning was found associated with increased rates of postpartum endometritis and blood loss (88). In settings where vaginal/uterine cleaning is not practiced, there is a contention that since evidence-based guidelines recommended the administration of narrow-range prophylactic antibiotics (89) before or during the surgery/CS such antibiotic is of putative neonatal benefit and sufficient enough to decrease the risk of infection as such initial cleansing of the vagina before the procedure may not be necessary. Further there is also recent evidence supporting the use of pre-incision, broad-spectrum antibiotics, which result in a lower rate of maternal morbidity with no disadvantage to the neonate (90). Additionally, applying vaginal cleansing agents would mostly likely predispose patients to other side effects, allergies, and irritation symptoms (91), therefore cleaning may not be required. Further and future investigation/clinical trials across populations are required to better understand the potential role of vaginal/uterine cleaning practice during CS procedures in influencing PPE.

### Postpartum vaginal microbiome, short interpregnancy interval, and its impact on multigravida

The microbiota of the female reproductive tract fluctuates throughout a women's lifespan and in response to circulating hormone levels. In women of reproductive age, rising estrogen levels promotes proliferation of vaginal epithelial cells and glycogen deposition which reduces the pH of the vaginal mucosa thus supporting the growth of Lactobacillus (92). Pregnancy is a unique phenomenon characterized by increasing placenta production of estrogen (93, 94) which potentiate lactobacillus drive with increasing gestational age (54). However, at the postpartum period there is a 100- to 1,000-fold dramatical decrease in steroid hormones (95) therefore any oestrogen-driven *Lactobacillus* sp. dominance of the vaginal microbiome during pregnancy is dynamically altered during the postpartum period to a vaginal microbiome depleted of *Lactobacillus* spp. and enriched with bacterial vaginosis associated species. This dynamism provides evidence that oestrogen is likely an important factor in shaping the composition of the vaginal microbiome, particularly during pregnancy (54, 96). Aside chemical modulation of the vaginal microbiome by steroid hormone. Duration, and cessation of lochia discharge is another important factor in shaping the vaginal microbial composition post-delivery (53). Lochia is an alkaline discharge that comprise of blood, serosa. This lochia-rich alkaline vaginal milieu during postpartum is a haven for the proliferation of *BVAB.* Discharge rates, volume, and cessation of lochia during post-delivery differs between subjects, but typically lasts about 6–8 weeks (97, 98) or more (99). This raises an important question of what period of time will any lochia driven BVAB vagitype revert to a Lactobacillus state after lochia cessation. To provide a clue, the work of Doyle and colleagues (2012), have showed that quantities of Lactobacillus were yet depleted in the vaginal microflora of women followed up for a period of 1 year instead a preponderance of BVAB was found in the microflora of women previously dominated with *L. crispatus* during pregnancy (59). The preponderance of BVAB vagitype (a notable driver of PTB) for a long period of time after lochia regression, may suggest why short interpregnancy interval or conception within a short period may pose a potential risk for preterm birth to multigravidas irrespective of age (20). Therefore, spacing subsequent pregnancy beyond 18 months may be required to reduce the risk of preterm birth or negative pregnancy outcome in multigravida. To keep up with this, data from a cohort of 148,544 pregnancies reveal that interpregnancy intervals shorter than 18 months are associated with higher risks of adverse pregnancy outcomes for women of all ages (20). To further support this notion, a recent study from a Finnish population observed that preponderance of *L. *crispatus** (compared to other species of Lactobacillus) was associated with only single birth per lifetime (primigravidas) compared to multigravidas with other lactobacillus spp or bacterial vaginosis associated vagitype that potentially increases the risk of premature delivery (100). The degree of protection conferred on the vaginal ecosystem is dependent on the predominant *Lactobacillus* species *L. crispatus* is considered optimal vaginal flora (101) as such the mechanism of transition to other vagitype during postpartum requires further investigation. Future study should investigate the period of restoration from the postpartum vaginal profile to the interpregnancy (normal) profile. A large longitudinal multicenter study is recommended to establish the composition of the postpartum vaginal microbiome accurately and to provide more insight into how a *Lactobacillus* profile is restored after lochia regression.

## Discussion/future consideration

The inconsistency in reports regarding the role of the vaginal microbiome in preterm birth highlights the current poor understanding of how the vaginal microbiome may promote healthy pregnancy outcomes. A detailed assessment of the relative contribution of delivery mode, birth practice and the postpartum vagitype will be critical for designing of better targeted strategies to restoring the reproductive health fitness of women of reproductive age. Large cohort studies should be designed to focus on how a woman is birthed versus her maternal microbiome and the corresponding infant microbiome of her child to further elucidate the relationship between maternal microbiome, obstetric outcome, and fetal outcome. Based on evidence discussed here, we think that there is a correlation between how a woman is birthed and what become her vaginal microbiome composition at pubertal and postpubertal age which may potentially impact pregnancy outcome. In addition, we hypothesize that this effect may extend into future reproduction to impact pregnancy outcome. In clinical settings, the occurrence of unnecessary cesarean section should reduce to abate the incidence of preterm birth and unhealthy microbiota during adulthood in post pubertal women. More large multicenter clinical trials are required to affirm the relative contribution of delivery practices (vaginal cleaning) during CS to postpartum endometritis. Recently, chlorhexidine and povidone- iodine are recommended vaginal cleansing antiseptics as they have minimal side effects, with low rates of allergies or irritation symptoms (87). Given the risk associated with CS potentiated PPE, comparative studies and randomized clinical trials would be helpful in provide more knowledge on the role of vaginal cleansing in CS potentiated PPE. This can lend new clue and useful insight to understanding the underlying cause of the disparity in the prevalence of PPE in high- and low-income countries. As studies continue to shed light on the potential role of the vaginal microbiome, delivery mode and delivery practices in preterm birth, they should engage the research community, clinicians, and the public to reassess the impact of delivery mode and delivery practices in negative pregnancy outcome such as preterm birth and postpartum complications.

## References

[1] Human Microbiome Project Consortium. A framework for human microbiome research. Nature. (2012) 486:215–21. 10.1038/nature11209 22699610 PMC3377744

[2] Hong KH Hong SK Cho SI Ra E Han KH Kang SB Analysis of the vaginal microbiome by next-generation sequencing and evaluation of its performance as a clinical diagnostic tool in vaginitis. Ann Lab Med. (2016) 36(5):441–9. 10.3343/alm.2016.36.5.441 27374709 PMC4940487

[3] Odogwu NM Olayemi OO Omigbodun AO. The vaginal microbiome of sub-saharan African women: revealing important gaps in the era of next-generation sequencing. PeerJ. (2020) 8:e9684. 10.7717/peerj.9684 32879794 PMC7441984

[4] Payne MS Newnham JP Doherty DA Furfaro LL Pendal NL Loh DE A specific bacterial DNA signature in the vagina of Australian women in midpregnancy predicts high risk of spontaneous preterm birth (the Predict1000 study). Am J Obstet Gynecol. (2021) 224(2):206.e1–206.e23. 10.1016/j.ajog.2020.08.034 32861687

[5] Fei L Zhou Y Zhu L Wang Z Ma L He Y Comparative metagenomic analysis of the vaginal microbiome in healthy women. Synth Syst Biotechnol. (2021) 6(2):77–84. 10.1016/j.synbio.2021.04.002 33997357 PMC8085786

[6] Smith SB Ravel J. The vaginal microbiota, host defence and reproductive physiology. J Physiol. (2017) 595(2):451–63. 10.1113/JP271694 27373840 PMC5233653

[7] Dalton E Castillo E. Postpartum infections: a review for the non-OBGYN. Obstet Med. (2014) 7(3):98–102. 10.1177/1753495X14522784 27512432 PMC4934978

[8] Brown RG Marchesi JR Lee YS Smith A Lehne B Kindinger LM Vaginal dysbiosis increases risk of preterm fetal membrane rupture, neonatal sepsis and is exacerbated by erythromycin. BMC Med. (2018) 16(1):9. 10.1186/s12916-017-0999-x 29361936 PMC5782380

[9] Bennett PR Brown RG MacIntyre DA. Vaginal microbiome in preterm rupture of membranes. Obstet Gynecol Clin North Am. (2020) 47(4):503–21. 10.1016/j.ogc.2020.08.001 33121642

[10] Odogwu NM Chen J Onebunne CA Jeraldo P Yang L Johnson S Predominance of atopobium vaginae at midtrimester: a potential indicator of preterm birth risk in a Nigerian cohort. mSphere. (2021) 6:e01261–20. 10.1128/mSphere.01261-20 33504666 PMC7885325

[11] Fettweis JM Serrano MG Brooks JP Edwards DJ Girerd PH Parikh HI The vaginal microbiome and preterm birth. Nat Med. (2019) 25(6):1012–21. 10.1038/s41591-019-0450-2 31142849 PMC6750801

[12] Hočevar K Maver A Vidmar Šimic M Hodžić A Haslberger A Premru Seršen T Vaginal microbiome signature is associated with spontaneous preterm delivery. Front Med. (2019) 6:201. 10.3389/fmed.2019.00201 PMC6746969 31552254

[13] Larsen B Hwang J. Mycoplasma, ureaplasma, and adverse pregnancy outcomes: a fresh look. Infect Dis Obstet Gynecol. (2010) 2010:521921. 10.1155/2010/521921 20706675 PMC2913664

[14] Jiao X Zhang L Du D Wang L Song Q Liu S. Alteration of vaginal microbiota in patients with recurrent miscarriage. J Obstet Gynaecol. (2022) 42(2):248–55. 10.1080/01443615.2021.1904851 34020581

[15] Pace RM Chu DM Prince AL Ma J Seferovic MD Aagaard KM. Complex species and strain ecology of the vaginal microbiome from pregnancy to postpartum and association with preterm birth. Med (New York, NY). (2021) 2(9):1027–49. 10.1016/j.medj.2021.06.001 PMC8491999 34617072

[16] Romero R Hassan SS Gajer P Tarca AL Fadrosh DW Bieda J The vaginal microbiota of pregnant women who subsequently have spontaneous preterm labor and delivery and those with a normal delivery at term. Microbiome. (2014) 2:18. 10.1186/2049-2618-2-18 24987521 PMC4066267

[17] Elovitz MA Gajer P Riis V Brown AG Humphrys MS Holm JB Cervicovaginal microbiota and local immune response modulate the risk of spontaneous preterm delivery. Nat Commun. (2019) 10(1):1305. 10.1038/s41467-019-09285-9 30899005 PMC6428888

[18] Dominguez-Bello MG Godoy-Vitorino F Knight R Blaser MJ. Role of the microbiome in human development. Gut. (2019) 68(6):1108–14. 10.1136/gutjnl-2018-317503 30670574 PMC6580755

[19] Stennett CA Dyer TV He X Robinson CK Ravel J Ghanem KG A cross-sectional pilot study of birth mode and vaginal microbiota in reproductive-age women. PLoS One. (2020) 15(4):e0228574. 10.1371/journal.pone.0228574 32236123 PMC7112195

[20] Schummers L Hutcheon JA Hernandez-Diaz S Williams PL Hacker MR VanderWeele TJ Association of short interpregnancy interval with pregnancy outcomes according to maternal age. JAMA Intern Med. (2018) 178(12):1661–70. 10.1001/jamainternmed.2018.4696. 30383085 PMC6583597

[21] Manuck TA Rice MM Bailit JL Grobman WA Reddy UM Wapner RJ Preterm neonatal morbidity and mortality by gestational age: a contemporary cohort. Am J Obstet Gynecol. (2016) 215(1):103.e1–103.e14. 10.1016/j.ajog.2016.01.004 26772790 PMC4921282

[22] Liu L Oza S Hogan D Chu Y Perin J Zhu J Global, regional, and national causes of under-5 mortality in 2000-15: an updated systematic analysis with implications for the sustainable development goals. Lancet. (2016) 388(10063):3027–35. 10.1016/S0140-6736(16)31593-8 27839855 PMC5161777

[23] Goldenberg RL Culhane JF Iams JD Romero R. Epidemiology and causes of preterm birth. Lancet. (2008) 371:75–84. 10.1016/S0140-6736(08)60074-4 18177778 PMC7134569

[24] Romero R Dey SK Fisher SJ. Preterm labor: one syndrome, many causes. Science. (2014) 345:760–5. 10.1126/science.1251816 25124429 PMC4191866

[25] Romero R Espinoza J Kusanovic JP Gotsch F Hassan S Erez O The preterm parturition syndrome. BJOG An Int J Obstet Gynaecol. (2006) 113:17–42. 10.1111/j.1471-0528.2006.01120.x PMC7062298 17206962

[26] Shimaoka M Yo Y Doh K Kotani Y Suzuki A Tsuji I Association between preterm delivery and bacterial vaginosis with or without treatment. Sci Rep. (2019) 9(1):509. 10.1038/s41598-018-36964-2 30679584 PMC6345902

[27] van Teijlingen NH Helgers LC Zijlstra-Willems EM van Hamme JL Ribeiro CMS Strijbis K Vaginal dysbiosis associated-bacteria Megasphaera elsdenii and Prevotella timonensis induce immune activation via dendritic cells. J Reprod Immunol. (2020) 138:103085. 10.1016/j.jri.2020.103085 32004804

[28] Robinson LS Perry J Lek S Wollam A Sodergren E Weinstock G Genome sequences of 15 Gardnerella vaginalis strains isolated from the vaginas of women with and without bacterial vaginosis. Genome Announc. (2016) 4(5):e00879–16. 10.1128/genomeA.00879-16 27688326 PMC5043544

[29] Kanayama N Terao T Horiuchi K. The role of human neutrophil elastase in the premature rupture of membranes. Asia Oceania J Obstet Gynaecol. (1988) 14:389–97. 10.1111/j.1447-0756.1988.tb00122.x 3263112

[30] Fortunato SJ Menon R Lombardi SJ. Role of tumor necrosis factor-alpha in the premature rupture of membranes and preterm labor pathways. Am J Obstet Gynecol. (2002) 187:1159–62. 10.1067/mob.2002.127457 12439495

[31] Shobokshi A Shaarawy M. Maternal serum and amniotic fluid cytokines in patients with preterm premature rupture of membranes with and without intrauterine infection. Int J Gynaecol Obstet. (2002) 79:209–15. 10.1016/S0020-7292(02)00238-2 12445984

[32] Helmig BR Romero R Espinoza J Chaiworapongsa T Bujold E Gomez R Neutrophil elastase and secretory leukocyte protease inhibitor in prelabor rupture of membranes, parturition and intra-amniotic infection. J Matern Fetal Neonatal Med. (2002) 12:237–46. 10.1080/jmf.12.4.237.246 12572592

[33] Perez-Muñoz ME Arrieta M-C Ramer-Tait AE Walter J. A critical assessment of the “sterile womb” and “in utero colonization” hypotheses: implications for research on the pioneer infant microbiome. Microbiome. (2017) 5:48. 10.1186/s40168-017-0268-4 28454555 PMC5410102

[34] Srinivasan S Liu C Mitchell CM Fiedler TL Thomas KK Agnew KJ Temporal variability of human vaginal bacteria and relationship with bacterial vaginosis. PloS One. (2010) 5(4):e10197. 10.1371/journal.pone.0010197 20419168 PMC2855365

[35] You YA Kwon EJ Choi SJ Hwang HS Choi SK Lee SM Vaginal microbiome profiles of pregnant women in Korea using a 16S metagenomics approach. Am J Reprod Immunol. (2019) 82:1. 10.1111/aji.13124 31134711

[36] Tamburini S Shen N Wu HC Clemente JC. The microbiome in early life: implications for health outcomes. Nat Med. (2016) 22:713–22. 10.1038/nm.4142 27387886

[37] Shao Y Forster SC Tsaliki E Vervier K Strang A Simpson N Stunted microbiota and opportunistic pathogen colonization in caesarean-section birth. Nature. (2019) 574:117–21. 10.1038/s41586-019-1560-1 31534227 PMC6894937

[38] Zhang Y Zhou J Ma Y Liu L Xia Q Fan D Mode of delivery and preterm birth in subsequent births: a systematic review and meta-analysis. PloS One. (2019) 14(3):e0213784. 10.1371/journal.pone.0213784 30870524 PMC6417656

[39] Soper DE. Infections of the female pelvis. In: Mandell GL Bennett JE Dolin R, editors. Mandell, douglas, and bennett's principles and practice of infectious diseases. 7th ed. Philadelphia, PA: Churchill Livingstone (2010). p. 1511–9.

[40] Salmanov AG Vitiuk AD Zhelezov D Bilokon O Kornatska AG Dyndar OA Prevalence of postpartum endometritis and antimicrobial resistance of responsible pathogens in Ukraine: results a multicenter study (2015-2017). Wiad Lek. (2020) 73(6):1177–83. 10.36740/WLek202006119 32723949

[41] Llagunes J Casanova I Reina C Carmona P Pirola A de Andrés J. Late-onset postpartum sepsis with septic shock and heart dysfunction. Revista Española de Anestesiología y Reanimación. (2011) 58(6):387–89. 10.1016/s0034-9356(11)70089-5 21797090

[42] Riad M Thottacherry E Crawley C Phillip-Abraham N Ibrahim F. Invasive group A streptococcal postpartum endometritis associated with multi-organ infarctions: an uncommon case presentation and literature review. Postgrad Med. (2020) 132(6):526–31. 10.1080/00325481.2020.1760031 32379557

[43] Axelsson D Brynhildsen J Blomberg M. Postpartum infection in relation to maternal characteristics, obstetric interventions and complications. J Perinat Med. (2018) 46(3):271–8. 10.1515/jpm-2016-0389 28672754

[44] Rouse CE Eckert LO Muñoz FM Stringer JSA Kochhar S Bartlett L Postpartum endometritis and infection following incomplete or complete abortion: case definition & guidelines for data collection, analysis, and presentation of maternal immunization safety data. Vaccine. (2019) 37(52):7585–95. 10.1016/j.vaccine.2019.09.101 31783980 PMC6891249

[45] Gonzalo-Carballes M Ríos-Vives MÁ Fierro EC Azogue XG Herrero SG Rodríguez AE A pictorial review of postpartum complications. Radiographics. (2020) 40(7):2117–41. 10.1148/rg.2020200031 33095681

[46] Kassebaum NJ Bertozzi-Villa A Coggeshall MS Shackelford KA Steiner C Heuton KR Global, regional, and national levels and causes of maternal mortality during 1990-2013: a systematic analysis for the global burden of disease study 2013. Lancet. (2014) 384(9947):956. 10.1016/S0140-6736(14)60696-6 24797575 PMC4255481

[47] Say L Chou D Gemmill A Tunçalp Ö Moller A-B Daniels J. Global causes of maternal death: a WHO systematic analysis. Lancet Glob Health. (2014) 2:e323–33. 10.1016/S2214-109X(14)70227-X 25103301

[48] Kawakita T Landy HJ. Surgical site infections after cesarean delivery: epidemiology, prevention and treatment. Matern Health Neonatol Perinatol. (2017) 3:12. 10.1186/s40748-017-0051-3 28690864 PMC5497372

[49] Rosene K Eschenbach DA Tompkins LS Kenny GE Watkins H. Polymicrobial early postpartum endometritis with facultative and anaerobic bacteria, genital mycoplasmas, and Chlamydia trachomatis: treatment with piperacillin or cefoxitin. J Infect Dis. (1986) 153(6):1028–37. 10.1093/infdis/153.6.1028 3701114

[50] Aldape MJ Bryant AE Stevens DL. Clostridium sordellii infection: epidemiology, clinical findings, and current perspectives on diagnosis and treatment. Clin Infect Dis. (2006) 43:1436. 10.1086/508866 17083018

[51] Jorup-Rönström C Hofling M Lundberg C Holm S. Streptococcal toxic shock syndrome in a postpartum woman. Case report and review of the literature. Infection. (1996) 24(2):164–7. 10.1007/BF01713330 8740114

[52] Masschaele T Steyaert S Goethals R. Leptrotrichia amnionii, an emerging pathogen of postpartum endometritis. Acta Clin Belg. (2018) 73(5):368–71. 10.1080/17843286.2017.1392090 29078736

[53] MacIntyre DA Chandiramani M Lee YS Kindinger L Smith A Angelopoulos N The vaginal microbiome during pregnancy and the postpartum period in a European population. Sci Rep. (2015) 5:8988. 10.1038/srep08988 25758319 PMC4355684

[54] Odogwu NM Onebunne CA Chen J Ayeni FA Walther-Antonio MRS Olayemi OO Lactobacillus crispatus thrives in pregnancy hormonal milieu in a Nigerian patient cohort. Sci Rep. (2021) 11(1):18152. 10.1038/s41598-021-96339-y 34518588 PMC8437942

[55] DiGiulio DB Callahan BJ McMurdie PJ Costello EK Lyell DJ Robaczewska A Temporal and spatial variation of the human microbiota during pregnancy. Proc Natl Acad Sci U S A. (2015) 112(35):11060–5. 10.1073/pnas.1502875112 26283357 PMC4568272

[56] Nunn KL Witkin SS Schneider GM Boester A Nasioudis D Minis E Changes in the vaginal microbiome during the pregnancy to postpartum transition. Reprod Sci. (2021) 28(7):1996–2005. 10.1007/s43032-020-00438-6 33432532 PMC8189965

[57] Severgnini M Morselli S Camboni T Ceccarani C Laghi L Zagonari S A deep look at the vaginal environment during pregnancy and puerperium. Front Cell Infect Microbiol. (2022) 12:838405. 10.3389/fcimb.2022.838405 35656029 PMC9152327

[58] Zhang X Zhai Q Wang J Ma X Xing B Fan H Variation of the vaginal microbiome during and after pregnancy in Chinese women. Genom Proteom Bioinform. (2022) 20(2):322–33. 10.1016/j.gpb.2021.08.013 PMC9684158 35093602

[59] Doyle R Gondwe A Fan Y-M Maleta K Ashorn P Klein N A Lactobacillus-deficient vaginal microbiota dominates postpartum women in rural Malawi. Appl Environ Microbiol. (2018) 84(6):e02150–17. 10.1128/AEM.02150-17 29305501 PMC5835753

[60] Susic DF Wang L Roberts LM Bai M Gia A McGovern E The P4 study: postpartum maternal and infant faecal microbiome 6 months after hypertensive versus normotensive pregnancy. Front Cell Infect Microbiol. (2022) 12:646165. 10.3389/fcimb.2022.646165 35198457 PMC8860159

[61] Severgnini M Morselli S Camboni T Ceccarani C Salvo M Zagonari S Gardnerella vaginalis clades in pregnancy: new insights into the interactions with the vaginal microbiome. PLoS One. (2022) 17(6):e0269590. 10.1371/journal.pone.0269590 35700195 PMC9197028

[62] Mackeen AD Packard RE Ota E Speer L. Antibiotic regimens for postpartum endometritis. Cochrane Database Syst Rev. (2015) 2015(2):CD001067. 10.1002/14651858.CD001067.pub3 25922861 PMC7050613

[63] Henderson J Carson C Redshaw M. Impact of preterm birth on maternal well-being and women's perceptions of their baby: a population-based survey. BMJ Open. (2016) 6(10):e012676. 10.1136/bmjopen-2016-012676 27855105 PMC5073632

[64] Daifotis HA Smith MM Denoble AE Dotters-Katz SK. Risk factors for postpartum maternal infection following spontaneous vaginal delivery complicated by chorioamnionitis. AJP Rep. (2020) 10(2):e159–64. 10.1055/s-0040-1709983 32426175 PMC7228805

[65] Myles TD Gooch J Santolaya J. Obesity as an independent risk factor for infectious morbidity in patients who undergo cesarean delivery. Obstet Gynecol. (2002) 100:959–64. 10.1016/s0029-7844(02)02323-2 12423861

[66] Seale AC Mwaniki M Newton CRJC Berkley JA. Maternal and early onset neonatal bacterial sepsis: burden and strategies for prevention in sub-saharan Africa. Lancet Infect Dis. (2009) 9:428–38. 10.1016/S1473-3099(09)70172-0 19555902 PMC2856817

[67] Henderson KJ Fuller KP Morosky CM. Manual exploration of the uterus as a risk factor for postpartum endometritis [251]. Obstet Gynecol. (2015) 125:81S. 10.1097/01.AOG.0000463228.81803.53

[68] van Dillen J Zwart J Schutte J van Roosmalen J. Maternal sepsis: epidemiology, etiology, and outcome. Curr Opin Infect Dis. (2010) 23:249–54. 10.1097/QCO.0b013e328339257c 20375891

[69] French LM Smaill FM. Antibiotic regimens for endometritis after delivery. Cochrane Database Syst Rev. (2004) 4:CD001067. 10.1002/14651858.CD001067.pub2 15495005

[70] Ahnfeldt-Mollerup P Petersen LK Kragstrup J Christensen RD Sørensen B. Postpartum infections: occurrence, healthcare contacts and association with breastfeeding. Acta Obstet Gynecol Scand. (2012) 91:1440–4. 10.1111/aogs.12008 23121089

[71] Guzman MA Prien SD Blann DW. Post-cesarean related infection and vaginal preparation with povidone-iodine revisited. Prim Care Update OB GYNS. (2002) 9(6):206–9. 10.1016/S1068-607X(02)00119-1

[72] Betrán AP Ye J Moller AB Zhang J Gülmezoglu AM Torloni MR. The increasing trend in caesarean section rates: global, regional and national estimates: 1990-2014. PLoS One. (2016) 11:e0148343. 10.1371/journal.pone.0148343 26849801 PMC4743929

[73] Petros PE. Re: global rising rates of caesarean sections. BJOG: Int J Obstet Gynaecol. (2021) 129(3):512–3. 10.1111/1471-0528.16889 34519160

[74] Betran AP Torloni MR Zhang JJ Gülmezoglu AM, WHO Working Group on Caesarean Section. WHO statement on caesarean section rates. BJOG. (2016) 123(5):667–70. 10.1111/1471-0528.13526 26681211 PMC5034743

[75] Caesarean section rates continue to rise, amid growing inequalities in access (who.int).

[76] Schwartz MA Wang CC Eckert LO Critchlow CW. Risk factors for urinary tract infection in the postpartum period. Am J Obstet Gynecol. (1999) 181(3):547–53. 10.1016/S0002-9378(99)70491-6 10486462

[77] Weinberg M Fuentes JM Ruiz AI Lozano FW Angel E Gaitan H Reducing infections among women undergoing cesarean section in Colombia by means of continuous quality improvement methods. Arch Intern Med. (2001) 161(19):2357–65. 10.1001/archinte.161.19.2357 11606152

[78] Betran AP Ye J Moller AB Souza JP Zhang J. Trends and projections of caesarean section rates: global and regional estimates. BMJ Glob Health. (2021) 6(6):e005671. 10.1136/bmjgh-2021-005671 34130991 PMC8208001

[79] Boerma T Ronsmans C Melesse DY Barros AJD Barros FC Juan L Global epidemiology of use of and disparities in caesarean sections. Lancet. (2018) 392(10155):1341–8. 10.1016/S0140-6736(18)31928-7 30322584

[80] Lyon JB Richardson AC. Careful surgical technique can reduce infectious morbidity after cesarean section. Am J Obstet Gynecol. (1987) 157(3):557–62. 10.1016/s0002-9378(87)80006-6 3307420

[81] Epstein RH. Get me out: A history of childbirth from the garden of eden to the sperm bank. New York: Norton (2010).

[82] Lakhi NA Tricorico G Osipova Y Moretti ML. Vaginal cleansing with chlorhexidine gluconate or povidone-iodine prior to cesarean delivery: a randomized comparator-controlled trial. Am J Obstet Gynecol MFM. (2019) 1(1):2–9. 10.1016/j.ajogmf.2019.03.004 33319753

[83] Ngonzi J Bebell LM Fajardo Y Boatin AA Siedner MJ Bassett IV Incidence of postpartum infection, outcomes and associated risk factors at mbarara regional referral hospital in Uganda. BMC Pregnancy Childbirth. (2018) 18:270. 10.1186/s12884-018-1891-1 29954356 PMC6022296

[84] Roeckner JT Sanchez-Ramos L Mitta M Kovacs A Kaunitz AM. Povidone-iodine 1% is the most effective vaginal antiseptic for preventing post-cesarean endometritis: a systematic review and network meta-analysis. Am J Obstet Gynecol. (2019) 221(3):261.e1–261.e20. 10.1016/j.ajog.2019.04.002 30954518

[85] Haas DM Pazouki F Smith RR Fry AM Podzielinski I Al-Darei SM Vaginal cleansing before cesarean delivery to reduce postoperative infectious morbidity: a randomized, controlled trial. Am J Obstet Gynecol. (2010) 202(3):310.e1-6. 10.1016/j.ajog.2010.01.005 20207251

[86] Weckesser A Farmer N Dam R Wilson A Morton VH Morris RK. Women's perspectives on caesarean section recovery, infection and the PREPS trial: a qualitative pilot study. BMC Pregnancy Childbirth. (2019) 19(1):245. 10.1186/s12884-019-2402-8 31307417 PMC6631540

[87] Haas DM Morgan S Contreras K Kimball S. Vaginal preparation with antiseptic solution before cesarean section for preventing postoperative infections. Cochrane Database Syst Rev. (2020) 4(4):CD007892. 10.1002/14651858.CD007892.pub7 32335895 PMC7195184

[88] Hamdy MA Taha OT Elprince M. Postpartum endometritis after uterine cleaning versus no cleaning in cesarean sections: randomized clinical trial. J Obstet Gynaecol Res. (2021) 47(4):1330–6. 10.1111/jog.14658 33438313

[89] Pinto-Lopes R Sousa-Pinto B Azevedo LF. Single dose versus multiple dose of antibiotic prophylaxis in caesarean section: a systematic review and meta-analysis. BJOG. (2017) 124(4):595–605. 10.1111/1471-0528.14373 27885778

[90] Lamont RF Sobel JD Kusanovic JP Vaisbuch E Mazaki-Tovi S Kim SK Current debate on the use of antibiotic prophylaxis for caesarean section. BJOG. (2011) 118(2):193–201. 10.1111/j.1471-0528.2010.02729.x 21159119 PMC3059069

[91] Rahimi S Lazarou G. Late-onset allergic reaction to povidone-iodine resulting in vulvar edema and urinary retention. Obstet Gynecol. (2010) 116(Suppl 2):562–4. 10.1097/AOG.0b013e3181e91fa3 20664455

[92] Boskey ER Cone RA Whaley KJ Moench TR. Origins of vaginal acidity: high D/l lactate ratio is consistent with bacteria being the primary source. Hum Reprod. (2001) 16:1809–13. 10.1093/humrep/16.9.1809 11527880

[93] Roy EJ Mackay R. The concentration of oestrogens in blood during pregnancy. J Obstet Gynaecol Br Emp. (1962) 69:13–7. 10.1111/j.1471-0528.1962.tb00002.x 14494691

[94] Siiteri PK MacDonald PC. Placental estrogen biosynthesis during human pregnancy. J Clin Endocrinol Metab. (1966) 26:751–61. 10.1210/jcem-26-7-751 4223909

[95] O'Hara MW Schlechte JA Lewis DA Wright EJ. Prospective study of postpartum blues. Biologic and psychosocial factors. Arch Gen Psychiatry. (1991) 48:801–6. 10.1001/archpsyc.1991.01810330025004 1929770

[96] Hyman RW Herndon CN Jiang H Palm C Fukushima M Bernstein D The dynamics of the vaginal microbiome during infertility therapy with in vitro fertilization-embryo transfer. J Assist Reprod Genet. (2012) 29:105–15. 10.1007/s10815-011-9694-6 22222853 PMC3270134

[97] Oppenheimer LW Sherriff EA Goodman JD Shah D James CE. The duration of lochia. Br J Obstet Gynaecol. (1986) 93(7):754–7. 10.1111/j.1471-0528.1986.tb07977.x 3755355

[98] Fletcher S Grotegut CA James AH. Lochia patterns among normal women: a systematic review. J Womens Health. (2012) 21(12):1290–4. 10.1089/jwh.2012.3668 23101487

[99] Muganyizi PS Kimario GF Rwegoshora FJ Paul PP Makins A. Impact of immediate postpartum insertion of TCu380A on the quantity and duration of lochia discharges in Tanzania. Contracept Reprod Med. (2021) 6(1):1. 10.1186/s40834-020-00145-2 33397504 PMC7783969

[100] Kervinen K Holster T Saqib S Virtanen S Stefanovic V Rahkonen L Parity and gestational age are associated with vaginal microbiota composition in term and late term pregnancies. EBioMedicine. (2022) 81:104107. 10.1016/j.ebiom.2022.104107 35759916 PMC9250009

[101] Witkin S. The vaginal microbiome, vaginal anti-microbial defence mechanisms and the clinical challenge of reducing infection-related preterm birth. BJOG: Int J Obstet Gynaecol. (2014) 122(2):149–275. 10.1111/1471-0528.13115 25316066

